# Supplementary material for: Evidence of leaky protection following COVID-19 vaccination and SARS-CoV-2 infection in an incarcerated population
Source: Nat Commun. 2023 Aug 19;14:5055. doi: 10.1038/s41467-023-40750-8 (PMC10439918; doi:10.1038/s41467-023-40750-8)
Supplement: Supplementary file 3 — Reporting Summary [file 41467_2023_40750_MOESM3_ESM.pdf]

nature portfolio

Corresponding author(s): Margaret L Lind
Albert I Ko

Last updated by author(s): Margaret L Lind

Reporting Summary

Nature Portfolio wishes to improve the reproducibility of the work that we publish. This form provides structure for consistency and transparency in reporting. For further information on Nature Portfolio policies, see our Editorial Policies and the Editorial Policy Checklist.

Statistics

For all statistical analyses, confirm that the following items are present in the figure legend, table legend, main text, or Methods section.

n/a	Confirmed	
☐	☒	The exact sample size (*n*) for each experimental group/condition, given as a discrete number and unit of measurement
☐	☒	A statement on whether measurements were taken from distinct samples or whether the same sample was measured repeatedly
☐	☒	The statistical test(s) used AND whether they are one- or two-sided<br>*Only common tests should be described solely by name; describe more complex techniques in the Methods section.*
☐	☒	A description of all covariates tested
☒	☐	A description of any assumptions or corrections, such as tests of normality and adjustment for multiple comparisons
☐	☒	A full description of the statistical parameters including central tendency (e.g. means) or other basic estimates (e.g. regression coefficient) AND variation (e.g. standard deviation) or associated estimates of uncertainty (e.g. confidence intervals)
☐	☒	For null hypothesis testing, the test statistic (e.g. *F*, *t*, *r*) with confidence intervals, effect sizes, degrees of freedom and *P* value noted<br>*Give P values as exact values whenever suitable.*
☒	☐	For Bayesian analysis, information on the choice of priors and Markov chain Monte Carlo settings
☒	☐	For hierarchical and complex designs, identification of the appropriate level for tests and full reporting of outcomes
☒	☐	Estimates of effect sizes (e.g. Cohen's *d*, Pearson's *r*), indicating how they were calculated

Our web collection on statistics for biologists contains articles on many of the points above.

Software and code

Policy information about availability of computer code

Data collection	We used R version 4.2.1 to process data.
Data analysis	We used R version 4.2.1 to perform all data analyses. Code generated to conduct the statistical analyses is available in the following repository: https://github.com/lindm89/CT_DOC_Dose_Effect_Vax.git.

For manuscripts utilizing custom algorithms or software that are central to the research but not yet described in published literature, software must be made available to editors and reviewers. We strongly encourage code deposition in a community repository (e.g. GitHub). See the Nature Portfolio guidelines for submitting code & software for further information.

Data

Policy information about availability of data

All manuscripts must include a data availability statement. This statement should provide the following information, where applicable:

- Accession codes, unique identifiers, or web links for publicly available datasets
- A description of any restrictions on data availability
- For clinical datasets or third party data, please ensure that the statement adheres to our policy

The data used in this study belongs to the Connecticut Department of Correction and cannot be shared publicly because of the presence of potential identifiable health and resident information. Qualified researchers may request for de-identified, patient level data by contacting the corresponding author with a detailed description of the research question and setting up a data use agreement with the Connecticut Department of Correction.

Research involving human participants, their data, or biological material

Policy information about studies with human participants or human data. See also policy information about sex, gender (identity/presentation), and sexual orientation and race, ethnicity and racism.

Reporting on sex and gender	The gender of residents is evaluated by two correctional officers at the time of intake based on the genitalia (observed during the intake strip search) and by governmental documents (passport, driver's license, and birth certificate). The study design did not include gender as an adjustment factor since male and female residents are housed in different facilities, and we accounted for facility in our model. Further, we did not conduct gender stratified analyses due to sample size limitations, especially for females who contributed 8% of the facility events during the delta predominate period and 12% of the facility events during the omicron predominate period. Resident characteristics by gender are reported in Supplemental Table 1.
Reporting on race, ethnicity, or other socially relevant groupings	Race and ethnicity are self identified within Connecticut Correctional Facilities. Within our study we adjusted for race and ethnicity as these social groups likely confound our associations of interest. We included a composite race and ethnicity factor comprised of three groups (non-Hispanic black, non-Hispanic white, and other). Please see Table 1 for breakdown.
Population characteristics	The study population was predominately male (89%) and the most common race/ethnicity was non-Hispanic Black (44.0%). The median age was 36 years old (IQR: 29-45 years).
Recruitment	Residents who were incarcerated during the study period (June 15, 2021 - May 10, 2022) were included if they spent at least one night in a cell with a roommate while incarcerated and were incarcerated for at least 14 days.
Ethics oversight	This project was determined to be a public health surveillance activity by the Yale University Institutional Review Board (ID: 2000031675).

Note that full information on the approval of the study protocol must also be provided in the manuscript.

Field-specific reporting

Please select the one below that is the best fit for your research. If you are not sure, read the appropriate sections before making your selection.

☐ Life sciences ☒ Behavioural & social sciences ☐ Ecological, evolutionary & environmental sciences

For a reference copy of the document with all sections, see nature.com/documents/nr-reporting-summary-flat.pdf

Behavioural & social sciences study design

All studies must disclose on these points even when the disclosure is negative.

Study description	We performed a retrospective, quantitative analysis using a rolling match cohort design.
Research sample	Our study sample comprised residents of Connecticut DOC facilities who were incarcerated between June 15, 2021, when Delta became the predominant variant in Connecticut according to sequenced clinical samples, and May 10, 2022. The sample was limited to residents who were incarcerated for at least 14 days, spent at least one night in a cell with a roommate and did not spend their whole incarceration in a restrictive housing unit. Residents were classified as having one of three SARS-CoV-2 exposures on a given day (cell exposure [n Delta period: 642, Omicron period: 702], cellblock exposure [n Delta period: 5,616, Omicron period: 5,980], or no documented exposure [n Delta period: 17,024, Omicron period: 13,464]). The included population was predominately male (90%), of middle age (mean age 37 years old [SD: 12 years]), and non-Hispanic black was the most common racial group (nH-black: 40%, nH-white: 32%, Hispanic: 27%, Other [Asian or American Indian]: 1%).<br><br>We excluded cellblock and no documented exposure events if a more proximal exposure occurred in the prior 14 days. We selected this sample because it was the most representative of the true population of people incarcerated within the CT DOC that allowed for our analysis aims. Specifically, we limited to people residing in cells with a roommate because they provided a population in which close SARS-CoV-2 exposures were easily identifiable (their cellmate tested positive). Within this population, we excluded the first 14 days of incarceration time because we did not have information on the person's recent exposure status (thus removing misclassification). While we strove to make this population as representative as possible, differences likely exist in which residents are housed within cells and dorms and, thus, our study's representativeness is specifically focused on residents of cells.
Sampling strategy	All cell and cellblock exposure events were selected and we randomly selected one event without documented exposure for each person if more than one occurred within a 14 day period. From the identified sample, we matched cell exposure events, cellblock exposure events, and events without documented exposure on facility and date. These data were retrospective in nature and the sample was determined by the analytical requirements. For this reason, no formal sample size calculation was performed. Though we recognize that we had limited precision for some of our effectiveness estimates (especially the cell exposure ones), the difference in effectiveness between cell and no documented exposure events was found to be significant. For this reason, we found our sample to be sufficient to provide initial evidence of leakiness.
Data collection	The data used in this analysis were collected as part of routine COVID-19 surveillance at the CT DOC and retrospectively queried for the CT DOC system for the analysis. Upon intake residents vaccination status (CTWiZ verified self report) was recorded in the surveillance database. Once incarcerated, residents vaccination offers and administered dose, COVID-19 tests (rapid antigen and RT-

	PCR), and test results are recorded by DOC staff and stored alongside the vaccination records data in the surveillance database. These data are collected for facility and surveillance purposes, not for research, and all data are collected without research questions in mind. However, the data was analyzed by MLL who knew the study aims at the time of analysis. Additionally, resident's demographic data (race, gender, and age) were recorded by DOC staff along with all within facility movement data in a facilities database.
Timing	The study period ran from June 15, 2021 through May 10, 2022.
Data exclusions	Among residents of the DOC during the delta predominate period of the analysis (June 15 - Dec. 12, 2021) 24 people were excluded because their whole incarceration period was within 90 days of a prior, recorded SARS-CoV-2 infection, 1,438 people were excluded because they spent less than 14 days within a DOC facility, 3,090 were excluded because they resided in dorms only (no time in cell), and 1,549 were excluded because they never had a roommate while in a cell. During the omicron predominate period we excluded 125 people their full incarceration time was within 90 days of a prior recorded positive, 1,007 people who were incarcerated for less than 14 days, 2,744 people who never resided in a cell, and 1,455 people who never had a roommate while in a cell.
Non-participation	Residents of the DOC are unable to opt out of surveillance data collection and, as this study used these data, there were no non-participants.
Randomization	This is an observational study, so no randomization occurred. We used a rolling matched cohort analysis to reduce bias, and accounted for additional confounding through model based adjustment and stratification.

Reporting for specific materials, systems and methods

We require information from authors about some types of materials, experimental systems and methods used in many studies. Here, indicate whether each material, system or method listed is relevant to your study. If you are not sure if a list item applies to your research, read the appropriate section before selecting a response.

Materials & experimental systems

n/a	Involved in the study
☒	☐ Antibodies
☒	☐ Eukaryotic cell lines
☒	☐ Palaeontology and archaeology
☒	☐ Animals and other organisms
☒	☐ Clinical data
☒	☐ Dual use research of concern
☒	☐ Plants

Methods

n/a	Involved in the study
☒	☐ ChIP-seq
☒	☐ Flow cytometry
☒	☐ MRI-based neuroimaging
